# Salt templated and graphene nanoplatelets draped copper (GNP-draped-Cu) composites for dramatic improvements in pool boiling heat transfer

**DOI:** 10.1038/s41598-020-68672-1

**Published:** 2020-07-20

**Authors:** Aniket M. Rishi, Satish G. Kandlikar, Anju Gupta

**Affiliations:** 1 Microsystems Engineering, Rochester Institute of Technology, 76 Lomb Memorial Drive, Rochester, NY 14623 USA; 2 Mechanical Engineering, Rochester Institute of Technology, 76 Lomb Memorial Drive, Rochester, NY 14623 USA; 3 Mechanical, Industrial and Manufacturing Engineering, University of Toledo, 2801 W. Bancroft St., Toledo, OH 43606 USA

**Keywords:** Mechanical engineering, Thermoelectric devices and materials, Engineering, Materials science

## Abstract

We demonstrate a novel technique to achieve highly surface active, functional, and tunable hierarchical porous coated surfaces with high wickability using a combination of ball milling, salt-templating, and sintering techniques. Specifically, using ball-milling to obtain graphene nanoplatelets (GNP) draped copper particles followed by salt templated sintering to induce the strength and cohesiveness to the particles. The salt-templating method was specifically used to promote porosity on the coatings. A systematic study was conducted by varying size of the copper particles, ratio of GNP to copper particles, and process parameters to generate a variety of microporous coatings possessing interconnected pores and tunnels that were observed using electron microscopy. Pool boiling tests exhibited a very high critical heat flux of 289 W/cm^2^ at a wall superheat of just 2.2 °C for the salt templated 3 wt% GNP draped 20 µm diameter copper particles with exceedingly high wicking rates compared to non-salt-templated sintered coatings. The dramatic improvement in the pool boiling performance occurring at a very low surface temperature due to tunable surface properties is highly desirable in heat transfer and many other engineering applications.

## Introduction

In the recent years, graphene-based composite coatings have been explored for their exceptional properties for various engineering applications such as corrosion protection^1–3^, electronics systems^4–7^, energy storage^8–11^ and biomedical industries^12,13^. Heat transfer applications require higher heat dissipation which can be achieved via surface active coatings and their higher thermal conductivities that assist in higher heat transfer rates while maintaining lower surface temperatures. Due to unique surface properties and higher thermal conductivity exhibited by graphene (~ 3,000–5,000 W/m K), there has been an ample interest in utilizing graphene-based coatings for heat transfer applications^14,15^. Additionally, various surface modification techniques such as chemical vapor deposition^16^, electrodeposition^17,18^, sintering^19,20^, and electroplating^21^ can be adopted to form highly surface-active graphene-based coatings with unique surface features that can impart properties to further improve the heat dissipation rates and overall performance.

Pool boiling is a two-phase heat transfer technique in which the properties of the surface coatings play a fundamental role in improving the heat transfer by constant liquid supply that inhibits the dry-out around a bubble which leads to the critical heat flux (CHF) condition. During CHF, a thin layer of vapor can form over the entire heater surface that may result in severe depreciation of heat transfer properties, specifically the CHF and heat transfer coefficient (HTC)^22,23^. Our previous studies indicate that surface wickability can dramatically improve the CHF and HTC by achieving very low wall superheats. We demonstrated this through electrodeposited graphene/copper based composite coatings possessing hierarchical porous network and highly wickable structures that yielded remarkable improvement in pool boiling^24^. Some of the noteworthy work performed in this field that have resulted in very high CHFs includes the use of micro/nanoscale coatings^25–27^, altering the heater surface wettability and wickability^28–30^, self-assembled structures with nanofluids as working fluids^31,32^.

This work presents a novel manufacturing approach to obtain surface active coatings via a combination of sintering, salt-templating, and ball milling techniques to yield graphene nanoplatelets (GNP)-draped-copper based sintered coatings. GNP were selected owing to their high surface area and thermal conductivity and their concentration in the coatings were varied with respect to copper particles (by weight). The surfaces displayed a hierarchical porous network with high wickability that resulted in high heat fluxes at very low surface temperatures. The porosity was further tuned by salt-templating during the sintering process. We hypothesize:

1. GNP draped copper particles obtained via ball milling may yield inhomogeneous coatings with optimum roughness that will further promote surface wetting and wicking properties for boiling applications.
2. Salt templating may lead to tunable porous coatings with hierarchical pores which will allow a continuous passage of bubbles and liquid during the pool boiling process.
3. Varying concentration of GNP and copper particle diameter will remarkably affect the surface morphology and related properties that are essential in achieving higher pool boiling performance compared to mono and multi-layered graphene.

## Results

### GNP-draped copper particles produced by ball milling

**Figure 1:**
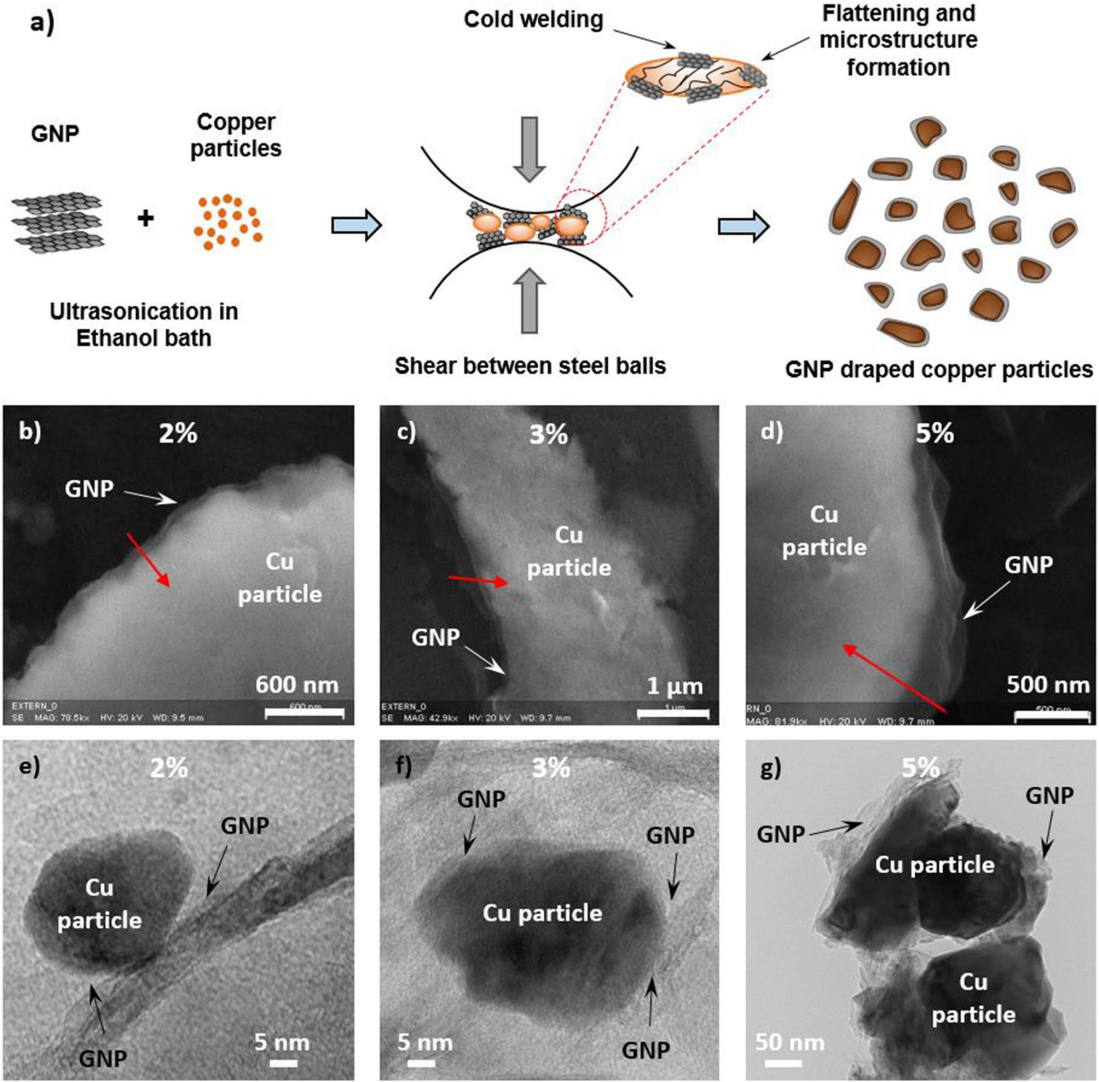
GNP draped copper particles via ball milling, Scanning and transmission electron microscopy images of 45 µm (**b**, **c**, **d**) and 20 µm (**e**, **f**, **g**) copper particles draped with 2% GNP, (**b**, **e**), (**b**) 3% GNP (**c**, **f**), and (**c**) 5% GNP (**d**, **g**).

Draped GNP copper particles were obtained by a solid-state powder processing technique which involved repeated cold welding, fracturing, and re-welding of copper particles in a high-energy ball mill as shown schematically in Fig. 1a. Smaller quantities of powdered mixtures of copper and varying GNP concentration were blended by loading into the ball milling chamber containing stainless-steel balls. The mixture was then agitated at higher speeds for a predetermined length of time. During this process, the copper and GNP particles are repeatedly flattened, cold welded, fractured and re-welded and the effect of collision between the constituent particles resulted in flattening and hardening of the composite particles as seen in Fig. 1. The collision of stainless-steel balls causes the entrapment of GNP and copper particles (Fig. 1a), as a result the GNP appears to drape around the individual copper particles. The force of impact plastically deforms the particles and results in fracture, subsequently work hardening of the particles. The severe plastic deformation also increases the surface-to-volume ratio of the particles. The repetitive events of ball to ball and ball to wall collisions throughout the milling period enables the cold welding which leads to the draping of GNP on copper particles. In this study, 15 min. of ball milling was followed by an hour of cooling to enable a short annealing cycle to promote the alloying and GNP draping phase. Annealing cycle also assisted in relieving internal stresses and defects of GNP which was further confirmed using Raman spectroscopy analysis (Fig. 3g) in the following section. This novel approach of implementing the annealing period during the ball milling process to yield GNP-draped-copper particles is schematically represented in Fig. 1a. The flattening of the particles during the ball milling process is validated via transmission electron microscope (TEM) images shown in Fig. 1e–g. Additionally, the pure shear between the milling balls prevented the agglomeration of the GNP^33^. The resultant GNP draped copper particles were then screen printed on copper test surfaces. Ball milling process promoted a considerable reduction in the particle size and homogenous dispersion of GNP on the sintered coatings confirmed via electron microscopy (see Supplementary Fig. S1 online).

To validate that the ball milling technique drapes GNP around copper particles, scanning and transmission electron microscope images were captured. Figure 1b–d show the scanning electron microscope (SEM) images of ball milled 45 µm copper particles at 2, 3, and 5 wt% GNP concentration. Higher concentration or wt% of GNP in the mixture resulted in draping of additional layers of GNP on copper particles which is shown in Fig. 1b–d, with red arrows indicating the direction of elemental analysis of the particles. A detailed elemental analysis is shown in supplementary information (see Supplementary Fig. S1 online) that shows an increment and stabilization of GNP and copper peaks in the direction of the arrow. The stabilization of GNP peak further confirmed the draping of GNP around the copper particles. The increment in GNP peak intensity resulting from draping of additional layers due to increased wt% of GNP was further confirmed using Raman spectroscopy analysis (discussed in the next sub-section). Transmission electron microscope (TEM) images (Fig. 1e–g) indicate subsequent reduction in particle sizes (~ 80–90% of the original particle size), indicating the drastic change from original circular shape to flattened structures with higher surface areas post ball milling. Initially, for 2% GNP, a smaller number of layers are draped around the copper particle (Fig. 1e). With increase in wt% of GNP from 2 to 5%, more layers of GNP get draped around the copper particles (Fig. 1f,g).

### Porous sintered GNP-draped copper surfaces produced by ball milling and salt templating

**Figure 2:**
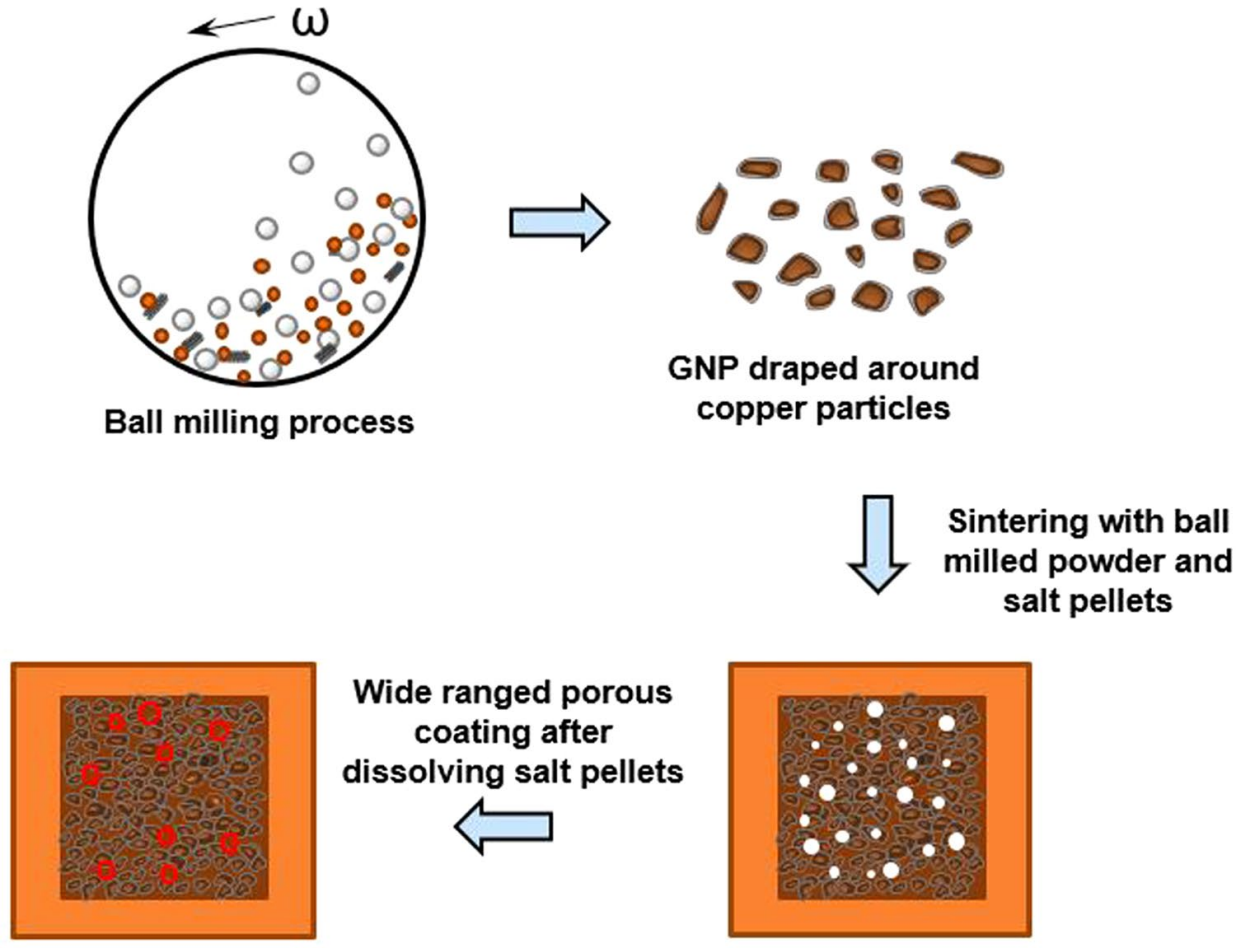
Schematic showing the development of coatings using salt templated sintering technique.

The GNP/copper coating was further improved by tuning the porosity and wickability of the surfaces using salt pellets as the templates during the sintering process. The schematic in Fig. 2 shows the overall salt templated sintering process. Precisely, sodium carbonate pellets were added to the ball milled GNP draped copper particles followed by sintering. Post sintering, the surface was rinsed thoroughly with distilled water to dissolve the salt pellets. Elemental analysis of the surfaces displayed no trace of salt on the surfaces before performing the pool boiling studies (see Supplementary Fig. S4 online). The thicknesses of the sintered coatings were measured using laser confocal microscope and average thickness of 65 ± 3 µm was obtained after sintering for all the coatings.

**Figure 3:**
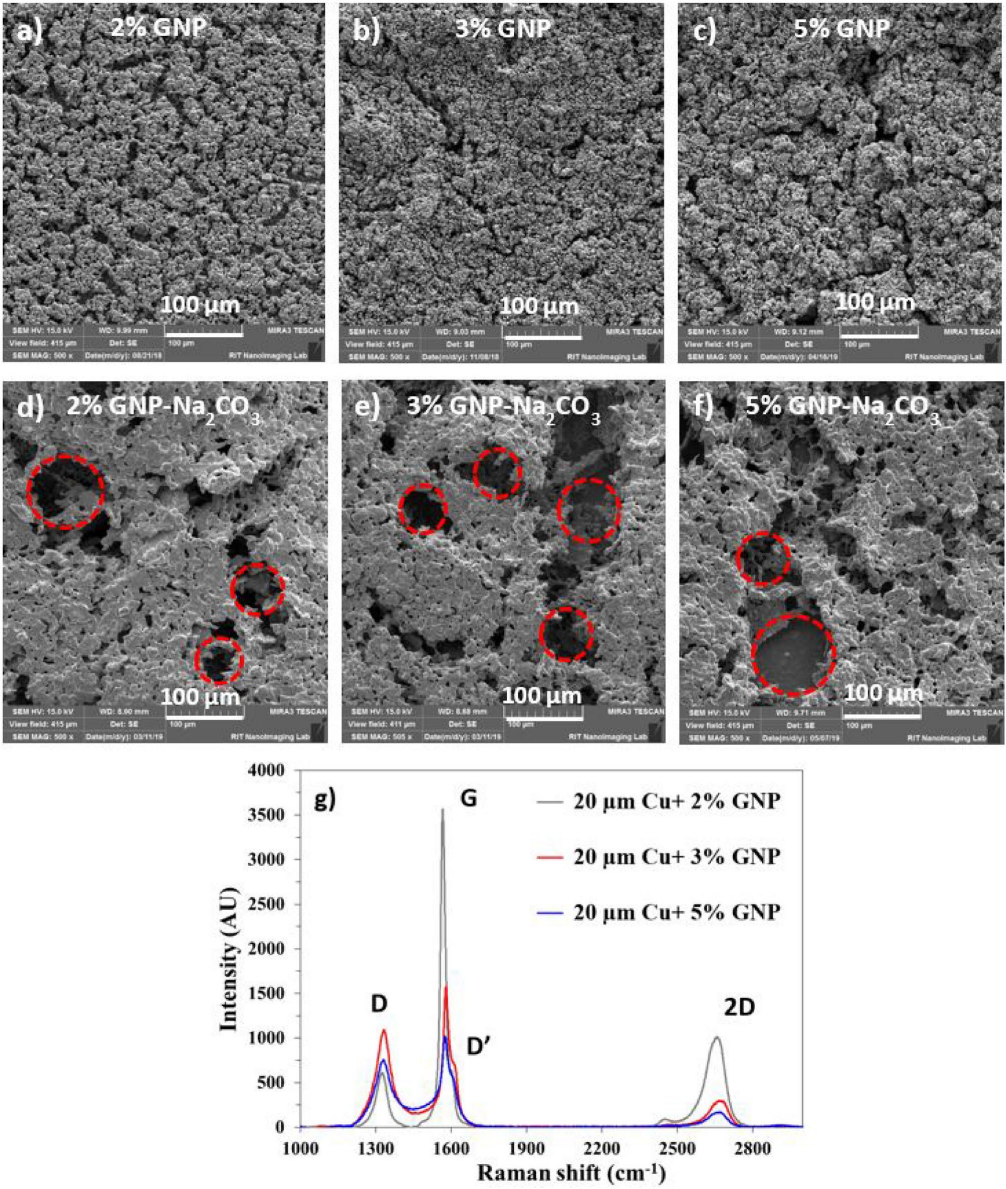
Comparison of scanning electron microscope (SEM) images of GNP draped 20 µm Cu coatings with (**a**) 2%, (**b**) 3%, and (**c**) 5% by weight GNP and (**d**) 2% GNP, (**e**) 3% GNP, and (**f**) 5% GNP via salt templating, and (**g**) Raman spectroscopy analysis of different wt.% GNP draped copper coatings.

SEM studies shown in Fig. 3 confirm the evolution of pores as a result of salt templating compared to the mere ball milled GNP-copper sintered surfaces. This was consistent for all the different weight ratios of GNP to copper particles. We established previously that the presence of hierarchical pores allows a continuous passage of bubbles and liquid via tunnel effect during the pool boiling process^30^. As shown in Fig. 3d–f a hierarchical porous network with pore diameters ranging from 2 µm to several hundred microns was achieved by salt templating. Coatings with 3 wt% GNP (Fig. 3e) exhibited an open porous network with the larger number of pores compared to 2% and 5% GNP surfaces.

Raman spectroscopy further confirmed the deposition of graphene nanoplatelets (GNP) layers on the copper particles and quantified the number of layers of GNP obtained as a result of varying concentrations of GNP. Figure 3g shows the typical G, D, and 2D peaks correspond to graphene correlating their in-plane vibrations of sp^2^-hybridized carbon atoms and degree of disorder of sp^3^-hybridized carbon structure^34,35^. The ratio of intensities of G peak to 2D peak (I_G_/I_2D_) yielded the number of deposited GNP layers for each GNP concentration. The intensity ratio I_D_/I_G_ of Raman spectra is typically used to identify the defects and damage degree of the graphene sheets. We observed I_D_/I_G_ ratio less than 1 for all the three samples indicating the less defects and good quality of deposited GNP. We observed an increment in the number of deposited GNP layers with increase in wt% of GNP. A maximum of ~ 7 layers were deposited for 5 wt% GNP, 3 wt% produced ~ 6 layers, while 2 wt% yielded ~ 4 layers of graphene.

For all the absorption peaks at D, G, and 2D, a slight shift of peaks toward right was observed owing to the application of higher wavelength of Laser (λ = 632.8 nm instead of λ = 514 nm) which has also been reported in the literature^36^. For all the samples, the absorption peaks for D and G bands were seen at ~ 1,335 cm^−1^ and ~ 1,580 cm^−1^. An additional small peak D’ at ~ 1,610 cm^−1^ was observed for all the three samples, which is attributed to intra-valley double resonant Raman process, where the defects provide missing momentum to satisfy the momentum conservation in a Raman scattering process^36^.

### Enhanced heat transfer properties during the pool boiling process: Effect of salt templated induced porosity and GNP concentration

**Figure 4:**
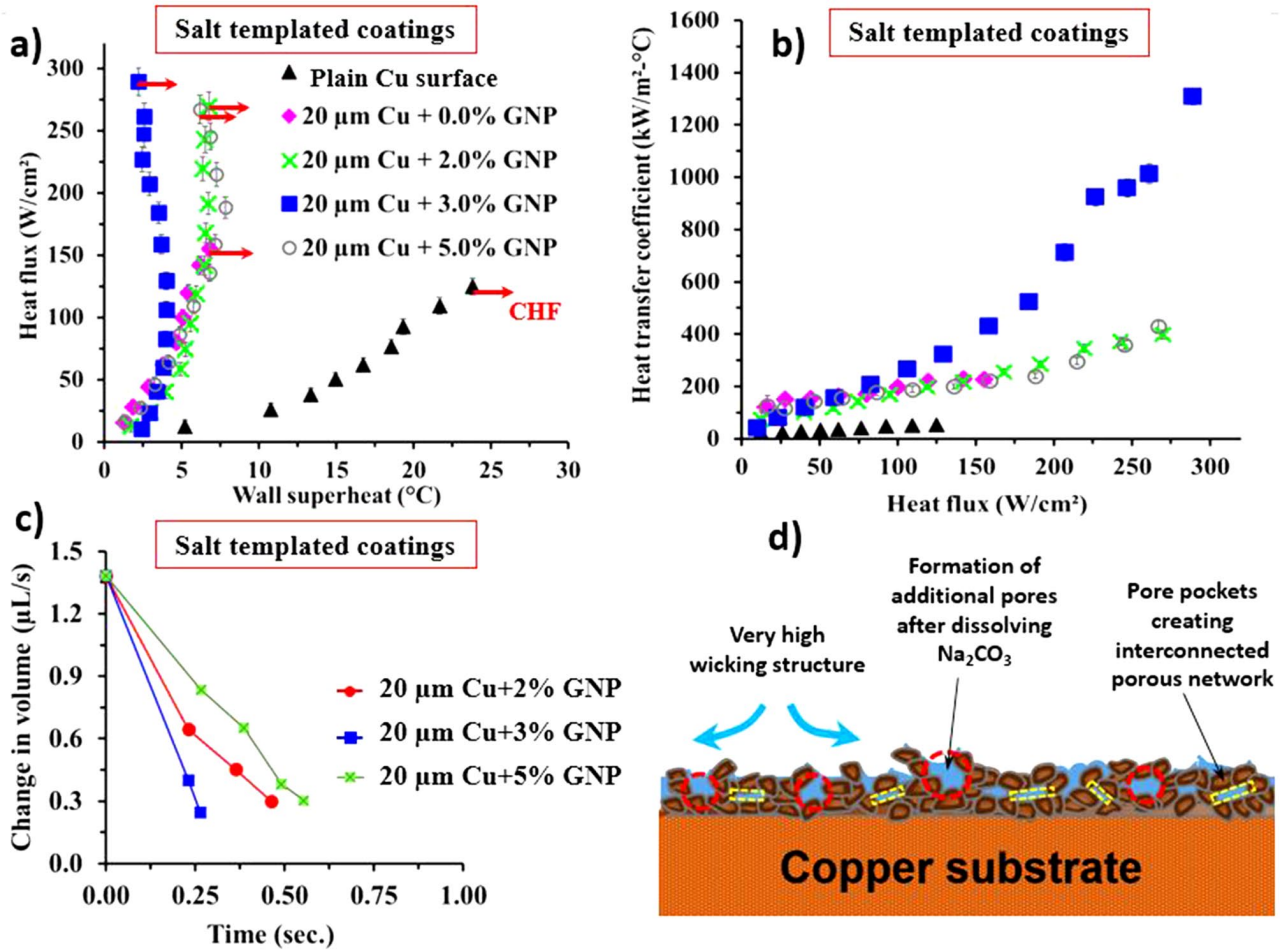
Comparison of effect of salt templating during sintering on pool boiling performance of 2%, 3%, and 5% GNP draped 20 µm Cu coatings showing (**a**) heat flux versus wall superheat depicting pool boiling regimes, (**b**) heat transfer coefficient versus heat flux summarizing heat transfer performance, (**c**) comparison of change in water droplet volumes, (**d**) schematic indicating the factors responsible for enhancement in pool boiling performance.

Pool boiling performance with regards the maximum heat dissipated by heater surface and the corresponding heat transfer coefficients was evaluated after characterization of the surfaces. A higher heat flux with lower wall superheat is desirable for engineering applications that involve very large heat transfer requirement in compact spaces. As a baseline, we performed the pool boiling study on a plain copper surface and achieved a CHF around 125 W/cm^2^ and HTC of 53 kW/m^2^°C^30^. Copper test surfaces used in this study were made of copper alloy 101 with 99.99% purity and were cleaned with IPA and distilled water before conducting the pool boiling tests and before coating via sintering. The average surface roughness of a plain copper surface was measured using laser confocal microscope after cleaning and for all the plain copper test surfaces, the average surface roughness (R_a_) was 1.3 µm and the static contact angle was 78°. Figure 4a,b show the comparison of pool boiling performance for 0%, 2%, 3%, and 5% GNP surfaces obtained via salt templating. A dramatic increment in CHF was observed for the combined ball milled and salt templated sintered surfaces. A maximum critical heat flux of 289 W/cm^2^ was attained for 20 µm copper-3% GNP surface, displaying approximately 131% enhancement in CHF compared to a plain copper surface. Furthermore, the wall superheat of just 2.2 °C was achieved indicating ~ 2,390% improvement in HTC in contrast to a plain copper surface. These are the highest CHF and HTC values reported in the pool boiling literature for graphene-based and porous coatings coated on a plain copper surface. For 0%, 2%, and 5% GNP coatings, CHF of 155 W/cm^2^, 269 W/cm^2^, and 267 W/cm^2^ were achieved respectively. The heat transfer coefficient for each heat flux was calculated (see Supplementary Eq. 4 online) for each surface after performing the pool boiling test and was plotted against the heat flux as shown in Fig. 4b. The maximum HTC of 1,314 kW/m^2^ °C was obtained for 20 µm Cu-3% GNP coating, while HTC of 227 kW/m^2^ °C, 399 kW/m^2^ °C, and 431 kW/m^2^ °C were achieved for 20 µm Cu-0%, 2%, and 5% GNP coatings (Fig. 4b), respectively.

A reduction in the wall superheat temperatures with increased heat flux, a phenomena termed as ‘ ‘Boiling Inversion” by Jaikumar and Kandlikar^37^ was also observed for their surfaces. This shift is attributed to the surface morphology of the coatings and increased active nucleation sites culminating from tunable porosity resulting from salt templating. However, owing to the complexity related to the combined effects of wettability, active nucleation site density, number of deposited graphene layers, and changes in the morphological structure, an in-depth study is required to understand the individual effects of each parameter.

To observe the effect of sintering via GNP-draped-copper particles and increased porosity via salt templating on wicking properties of the coatings, we measured wettability and wickability of the coated surfaces using a sessile water droplet method before conducting the pool boiling experiments. After measuring the contact angles of these surfaces using a Goniometer, we found that all the coatings were superhydrophilic (0° contact angle). Thus, the wicking rates of the coatings were used for the comparison. We found that salt templated 20 µm Cu-3% GNP surface yielded the highest wicking rate indicating the highest wicked volume (Fig. 4c) as compared to salt templated 20 µm Cu-2% and 5% GNP surfaces. Figure 4c compares the change in water droplet volume with respect to time for all the surfaces. Higher wickability of the coatings allows a continuous liquid supply to the nucleation cavities during boiling, thereby delaying the vapor layer formation and enhancing the critical heat flux of the coatings. Several reports^38–40^ have shown that thermal conductivity of a few layered graphene (2–4 layers) is in the range of ~ 2,300 W/m K to ~ 3,000 W/m K (as against 3,000 W/m K to 5,000 W/m K for a single layer of graphene). And reduces to ~ 2000 W/m K with increase in the number of deposited graphene layers (greater than 4 layers). It is postulated that the draping of GNP on individual copper particles obtained via ball milling increased the overall thermal conductivity of the coatings. And since the pool boiling performance is primarily governed by the thermal conductivity of the coating, this increment in thermal conductivity of the coatings contributed in the enhancement of the CHF.

A detailed morphological analysis of the coatings reveals inhomogeneous and irregular porous surface morphology as a result of combined ball milling and sintering with salt templating (Figs. 3e–g, and 6). Ball milling induced additional rectangular pores termed as “pore pockets” as shown in Fig. 6b. Salt templating and sintering methods provided supplementary pores with a wide range of porosity ranging from ~ 2 to 200 µm. To further understand the effect of these additional pores on nucleation activity, application of Hsu’s model^41^ to the pool boiling results can support in estimating the cavity sizes that nucleate at different wall superheat temperatures. This model can also assist in determining the cavity sizes that nucleate at the point of “Boiling Inversion” and the cavity sizes which act as liquid reservoirs. The range of active nucleation cavity sizes is determined by the following equation:

1 $$\left[ {R_{c,\max } ,R_{c,\min } } \right] = \frac{{\delta_{t} C_{2} }}{{2C_{1} }}\left[ {\frac{{\Delta T_{sat} }}{{\Delta T_{sat} + \Delta T_{sub} }}} \right] \times \left[ {1 \pm \sqrt {\frac{{1 - 8C_{1} \sigma T_{sat} \left( {\Delta T_{sat} + \Delta T_{sub} } \right)}}{{\rho_{v} h_{fg} \delta_{t} (\Delta T_{sat} )^{2} }}} } \right]$$

where $C_{1} = 1 + \cos \theta_{r}$ and ${ }C_{2} = \sin \theta_{r}$. $R_{c,max} { }and{ }R_{c,min}$ are maximum and minimum radii of the nucleation cavities, $\theta_{r}$ is the receding contact angle, $\delta_{t}$ is thermal boundary layer thickness (m), $\Delta T_{sat}$ is the wall superheat temperature ($\Delta T_{sat} = T_{surface} - T_{sat}$) (K), $\Delta T_{sub}$ is the subcooled temperature (K), $\sigma$ represents the surface tension of water at saturation temperature (N/m), $\rho_{v}$ is the vapor density (kg/m^−3^), and $h_{fg}$ represents the latent heat of vaporization (J/kg).

**Figure 5:**
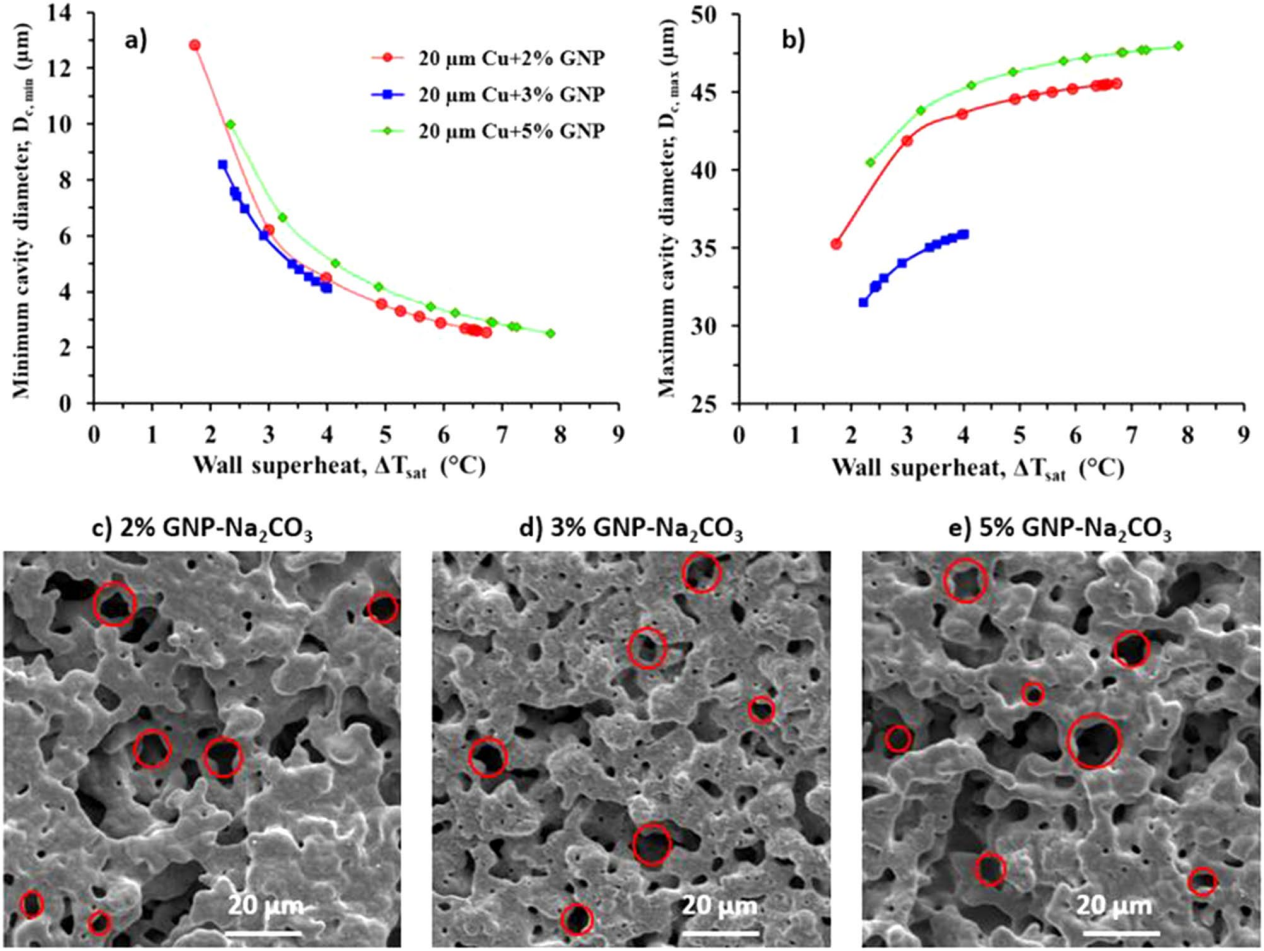
Range of active nucleation cavities for 2%, 3%, and 5% GNP coatings showing (**a**) minimum cavity diameters, (**b**) maximum cavity diameters as a function of wall superheat temperature using Hsu’s model, (**c**), (**d**), and (**e**) SEM images at 2 k × magnification confirming the availability of wide range of porous network in the estimated range of diameters.

Figure 5a,b show the minimum and maximum diameters of the active nucleation cavities for all three coatings. The receding contact angle for each surface was measured at multiple nucleation sites using a high-speed camera and the average value was considered while calculating the range of active nucleation cavities. The plots show the range of possible nucleation cavity diameters, 45.5 µm to 2.6 µm for 2% GNP coating, 31.5 µm to 8.5 µm for 3% GNP coating, and 47.3 µm to 3.3 µm for 5% GNP coating. These diameters are estimated using Eq. (1) and similar to our previous work^19^, the HTC of 6,000 W/m^2^ °C was used assuming a linear thermal boundary layer during the calculation of boundary layer thickness for each surface. Figure 5c–e as well as Fig. 3d–f demonstrate the morphologies of the salt templated sintered coatings confirming the pores with different range of cavity sizes. These cavity sizes are within the predicted nucleation range of Fig. 5a,b. When the pool boiling curve (see Fig. 4a) begins to shift to the left, the effects arising from additional cavities is amplified. Consequently, the HTC increases significantly due to the increased contribution from the rapid nucleation activity.

**Figure 6:**
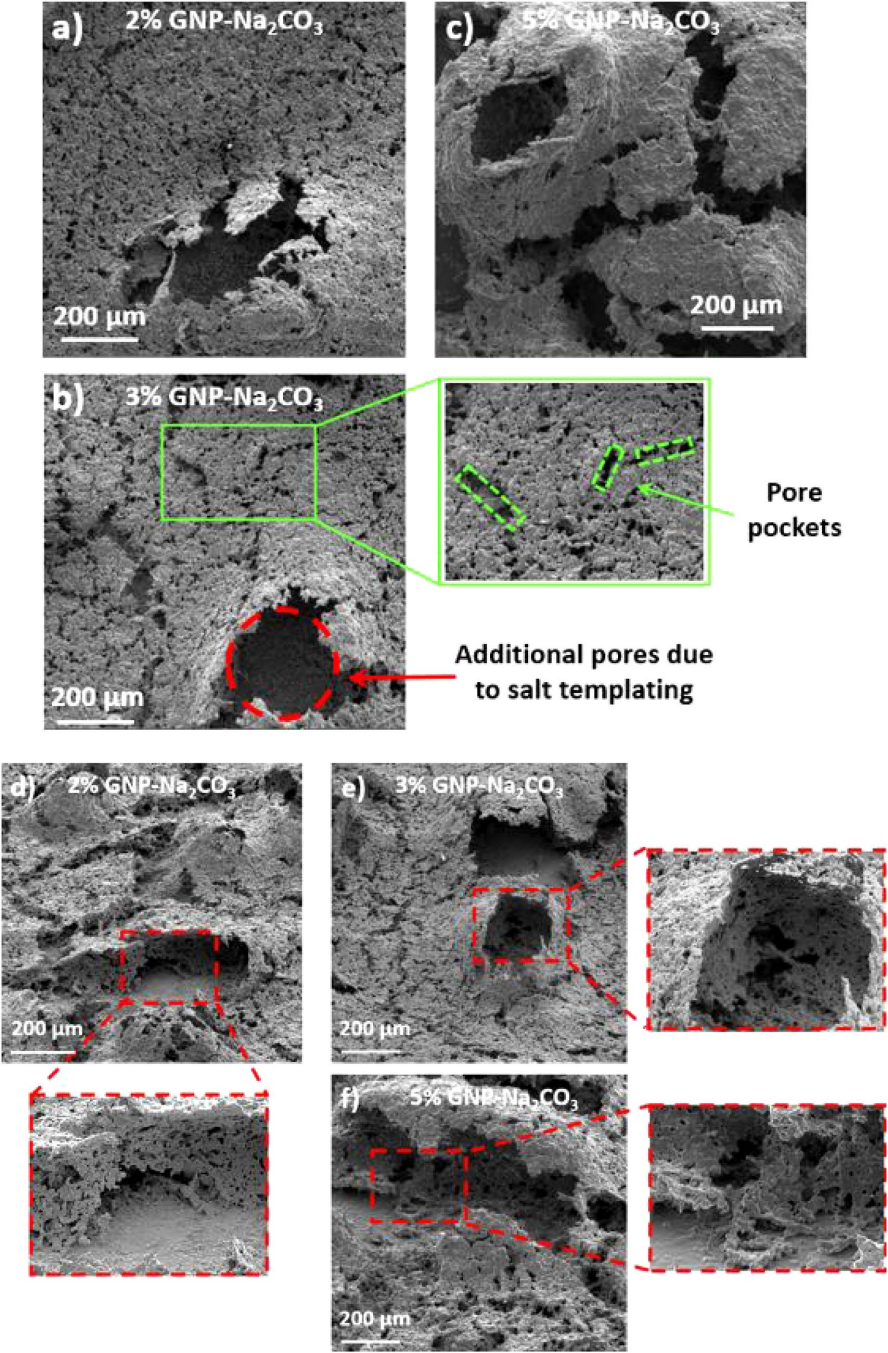
Scanning electron microscope images at 200 × for salt templated GNP draped Cu coatings (20 µm Cu particles) (**a**) 2% GNP, (**b**) 3% GNP, and (**c**) 5% GNP, stage tilted scanning electron microscope images for salt templated GNP draped Cu coatings (**d**) 2% GNP, (**e**) 3% GNP, and (**f**) 5% GNP.

From the plot shown in Fig. 5a, it is observed that as the wall superheat increases, smaller nucleation cavities activate, resulting in significant enhancement in HTC due to the amplified contribution from the rapid nucleation activity. It is proposed that the cavities outside the range of active nucleation site diameter range act as liquid reservoirs and liquid supply sites (Fig. 6a–f). The larger pores resulted from the salt templating (Fig. 6a–c) and micro, nanoscale tunnels resulted from sintering (Fig. 6d–f) thus provide a continuous liquid supply to the nucleating cavities, thereby increasing both CHF and HTC. Compared to 2% and 5% GNP surfaces (Fig. 6a,c), in case of 3% GNP coating, additional liquid pathways are developed on the coating termed as “pore pockets” (length: 50–150 µm and width: 10–50 µm) (see Fig. 6b). Combination of all these factors resulted in achieving the lowest wall superheat temperature (2.2 °C) and the highest CHF (289 W/cm^2^) for 3% GNP coating.

To further understand the effect of salt templated coatings on bubble dynamics, high-speed images for 2%, 3%, and 5% GNP salt templated coatings were obtained at a heat flux of ~ 10 W/cm^2^ using a Photron Fastcam high-speed camera. As a baseline, the bubble dynamics was captured for the plain copper surface. The total time required for a single bubble to nucleate, grow, and depart was 13.25 ms and a bubble departure diameter of 2.1 mm was attained for a plain copper surface. Due to the presence of hierarchical porous structures on the salt templated sintered coatings, rapid bubble growth was observed for all coatings containing GNP wt% at varying ratios. A smallest bubble departure diameter of 0.452 mm at the shortest departure time of 2.25 ms was recorded for the 20 µm Cu-3% GNP salt templated coating (see Supplementary Fig. S8 online). While departure diameters and time of 0.51 mm, 3 ms and 0.554 mm, 2.75 ms were observed for 20 µm Cu-2% GNP and 20 µm Cu-5% GNP salt templated coatings, respectively. Rapid bubble departures in short durations for the salt templated coatings resulted in high heat transfer performances.

Total 3 repetitive pool boiling tests were performed on the best performing 20 µm Cu-3% GNP salt templated coating. A small deterioration in the pool boiling performance was observed at the end of the third repetitive pool boiling test, yielding the CHF of 279 W/cm^2^ at a wall superheat of 4.9 °C. This can be minimized by stopping the pool boiling test before the surface hits the CHF. The change in wetting behavior was observed for the coating after 3 repetitive boiling tests. A similar change in wetting properties was also observed for our previous work^28^ involving graphene-based coatings and is attributed to the removal of oxygen groups from graphene due to repetitive boiling. Despite small reduction in the pool boiling performance, strong adhesion between the coating and the substrate was observed. Nevertheless, further studies are warranted to observe the effect of additional repetitive tests on the pool boiling performance of the coatings.

## Discussions

This work presents a novel approach of obtaining highly surface-active composite coatings with tunable properties by combining ball milling, sintering and salt-templating techniques. These coatings demonstrated a significant enhancement in the pool boiling heat transfer performance by yielding the highest critical heat flux (289 W/cm^2^) and the heat transfer coefficient (1,314 kW/m^2^ °C), compared to similar surfaces reported in the literature. These high CHF and HTC values are desirable for effective thermal management in many applications including electronics cooling, reboilers, and heat exchangers. Our extensive studies indicated that an alternate 15 min. ball milling and 1 h. cooling cycles implemented during the ball milling process draped higher thermal conductivity GNP on individual copper particles (GNP ~ 2000–3,000 W/m K and copper ~ 391 W/m K) which ultimately enhanced wicking and thermal properties of the coatings, thereby dramatically improving the pool boiling performance. We also attribute this dramatic enhancement in heat transfer performance to the salt templating via addition of Na_2_CO_3_ pellets during sintering process and eliminating them after sintering to achieve a wide range of porous network. Wide range of available porous network and pore openings act as liquid retention reservoirs to provide a continuous liquid supply to the nucleation sites which delays the vapor blanket formation. As indicated in schematic 4 d), unique properties of GNP-draped-copper particles and a subsequent formation of microporous coating with salt templating using Na_2_CO_3_, optimized the factors responsible for higher pool boiling performance. A systematic study of the effects of each technique i.e. mere sintering and ball milling followed by sintering was conducted initially and their performance was compared with salt templated sintered coatings. The effect of copper particles sizes during the ball milling was also explored to optimize the pool boiling performance and provide the basis for developing ball milled salt templated sintered coatings.

**Figure 7:**
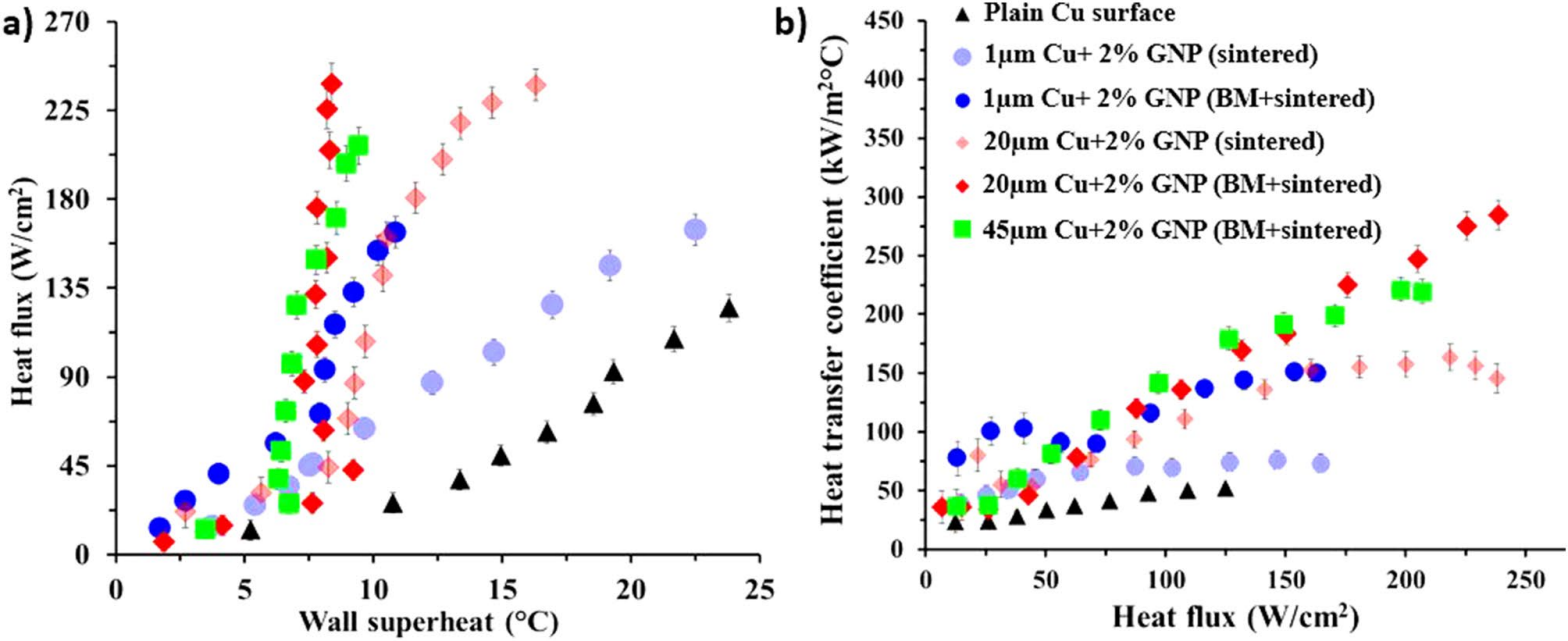
Comparison of the effect of ball milling and effect of copper particle size during GNP draping on pool boiling performance showing (**a**) heat flux versus wall superheat depicting pool boiling regimes, (**b**) heat transfer coefficient versus heat flux summarizing heat transfer performance.

Preliminary studies were performed to compare the effect of sintering with ball milled GNP/copper particles and sintering with GNP/copper particles mixture (without ball milling). During initial experiments, all the sintered coatings were developed without salt templating. The coatings with these two methods were formed using 1 µm copper, and 20 μm copper particles/2% GNP and their pool boiling performance was compared. The coatings developed by only sintering technique (labeled as sintered in Fig. 7) attained a very high wall superheat temperature (16.3 °C and 22.5 °C) and a CHF of 237 W/cm^2^ and 164 W/cm^2^ for 20 µm copper-2% GNP and 1 µm copper-2% GNP coatings, respectively (Fig. 7a). Compared to that, ball milled followed by sintered coatings (labeled as BM + sintered in Fig. 7) of 20 µm copper-2% GNP and 1 µm copper-2% GNP yielded very low wall superheat temperatures (8.4 °C and 10.8 °C) with a CHF of 239 W/cm^2^ and 164 W/cm^2^ (Fig. 7a), respectively. The electron microscopy confirmed the development of a unique morphological structure (see Supplementary Fig. S2 online) as a result of the ball milling technique which resulted in higher pool boiling performance. This indicated that the ball milling followed by sintering is advantageous for achieving higher pool boiling efficiencies.

The effect of varying copper particle sizes during the ball milling was also investigated using three different copper particle sizes—1 µm, 20 µm, and 45 µm while keeping GNP concentration to 2 wt%. The 2 wt% GNP concentration was selected for all the coatings in this work based on our previous findings^30^. A detailed comparison of surface morphologies obtained with varying copper particle sizes, and the quantification of number of GNP layers for each is provided in the supporting information (see Supplementary Fig. S3 online). With increased particle sizes, a continuous improvement in the pool boiling performance was achieved, however, a drop in the pool boiling performance was observed with 45 µm copper particles (see Fig. 7). Figure 7a shows a highest critical heat flux of 239 W/cm^2^ at a wall superheat of 8.3 °C for ball milled 2% GNP-20 µm Cu surface, which represents a dramatic increase in the CHF by ~ 91%. Owing to the achievement of the highest performance with 20 µm copper particles, all the salt-templated sintered coatings were developed using 20 µm copper particles.

The calculated heat transfer coefficients of the surfaces were compared to heat fluxes as shown in Fig. 7b. A maximum heat transfer coefficient of 285 kW/m^2^ °C was obtained for 2% GNP draped 20 µm copper coating, indicating an enhancement by ~ 438% compared to a plain copper surface. Additionally, a heat transfer coefficient of 73 kW/m^2^ °C was achieved for merely sintered 1 µm copper-2% GNP coating, while for ball milled 2% GNP-1 µm copper coating HTC was 150 kW/m^2^ °C. Overall, we observed a massive enhancement in heat transfer coefficient after the implementation of the ball milling of GNP and copper particles. Availability of a very effective liquid supply pathway and higher wicking owing to uniform distribution of GNP throughout the porous network are responsible for this huge enhancement in HTC. Higher heat transfer coefficients are indicative of extremely high heat transfer efficiencies.

The enhancement mechanism of the ball milled followed by sintered coatings is established in this section. Due to the constant 2 wt% GNP used with 1, 20, and 45 µm copper particles, the number of layers of graphene deposited on the surfaces remain unchanged (see Supplementary Fig. S3-d online). Alteration in surface properties such as wickability, porosity, and overall morphology of the coatings arise due to the processing method such as ball milling or sintering resulted in variation in the heart transfer properties. Microscopically, higher CHF and HTC are attributed to the formation of optimum sized porous pockets and the nano-sized cavities which act as pathways of liquid supply in the nucleation cavities. Owing to their surface activity and consequent boiling performance, 20 µm copper particles draped with 2% GNP were further implemented in salt-templated sintered coatings.

**Figure 8:**
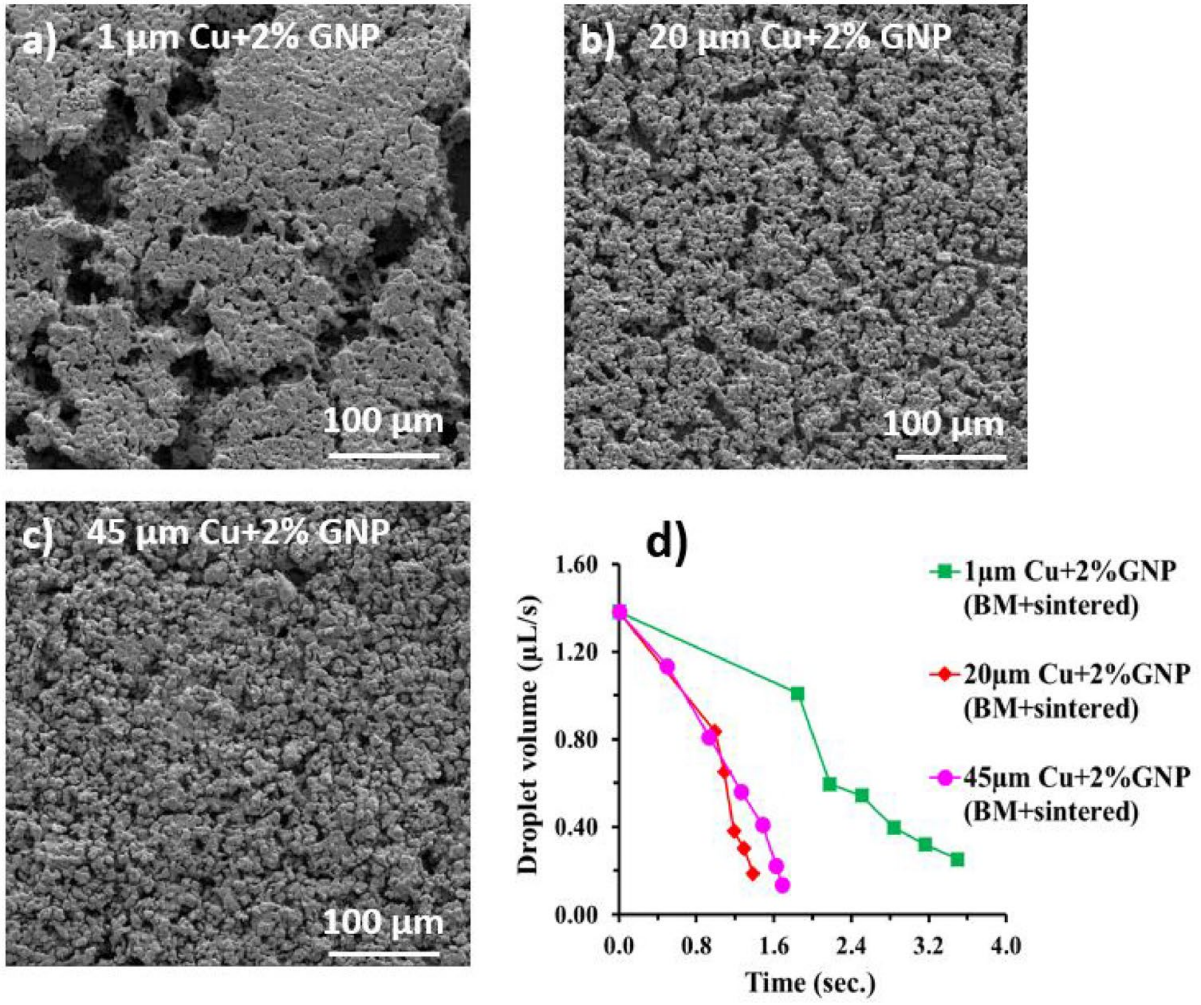
Scanning electron microscope (SEM) images at 500 × magnification of GNP draped Cu coatings (**a**) ball milled and sintered (BM + sintered) 1 µm Cu-2% GNP, (**b**) BM + sintered 20 µm Cu-2% GNP, (**c**) BM + sintered 45 µm Cu-2% GNP, (**d**) comparison of change in water droplet volume over time.

Our previous works have confirmed that the liquid spreading on wickable surfaces delay the formation of vapor layer, while nucleation cavities ensures the necessary liquid resupply to the surfaces. Owing to the higher wettability of GNP and copper particles, the resultant surfaces measured a contact angle of 0° indicating the superhydrophilicity of the coatings. The wickability and wicking rates of 1, 20, and 45 µm Cu-2% GNP surfaces were performed using sessile water droplet method. Figure 8d shows the comparison of change in water droplet volume with respect to time for all three surfaces. We found that the ball milled 2% GNP-20 µm Cu surface yielded the highest wicking rate indicating the highest wicked volume as compared to the ball milled 2% GNP 1 µm and 45 µm copper surfaces. Thus, we established that higher wickability is one of the factors responsible for the increment in CHF and reduction in wall superheat temperature of the ball milled 2% GNP-20 µm Cu surface.

To comprehend the role of morphological structure in enhancing the overall pool boiling performance, we performed an in-depth morphological analysis on 1, 20, and 45 µm Cu-2% GNP coatings. We observed that there is an optimum size of pore pockets that is responsible for enhancing the effective liquid pathway and a maximum vapor departure. For example, in case of 2% GNP-1 µm copper surface, owing to the continuous collision between stainless steel balls and the powder during the ball milling process, the size of the copper particles reduced further, and agglomeration of these particles was observed after sintering (Fig. 8a), which led to the formation of huge pore pockets with a length of ~ 92 µm and a width of ~ 24 µm after sintering process (Fig. 8a, calculated using ImageJ software). This caused early bubble coalescence and reduced the CHF and HTC for 2% GNP-1 µm copper surface. We believe that the reduction in particle size after the ball milling process for 2% GNP-20 µm copper surface was optimum. This surface generated pore pockets of ~ 32 µm long and ~ 7.4 µm wide (Fig. 8b, calculated using ImageJ software). While for 2% GNP-45 µm copper surface, the pore pockets of ~ 28 µm long and ~ 9 µm wide were formed (Fig. 8c).

In addition to the pore pocket dimensions, we believe that the number of pore pockets also play a crucial role in deciding the pool boiling performance and efficiency of the heater surface. For the SEM images shown in Fig. 8, the view field is 415 µm at a magnification of 500X. Thus, for this same view field in all the three images (Fig. 8a–c), maximum of 17 pore pockets were observed for 2% GNP-20 µm Cu surface, as against 6 and 8 for 2% GNP-1 µm and 45 um Cu surfaces, respectively. Owing to the formation of a large number of optimum sized interconnected porous pockets, 20 µm copper-2% GNP coated surface yielded the highest performance as compared to 1 µm and 45 µm copper-2% GNP surfaces. This further validates the usage of the initial size of 20 µm copper particles for developing the salt templated sintered coatings.

Lastly, the effect of salt templating on enhanced boiling performance is evident from Figs. 4 and 7. For instance, a CHF of 270 W/cm^2^ at a wall superheat of 6.8 °C was achieved for salt templated and ball milled 2% GNP-20 µm copper coating, rendering ~ 13% enhancement in CHF and ~ 52% enhancement in HTC when compared with non-salt templated ball milled 2% GNP-20 µm copper coating. The additional enhancement is primarily due to formation of porous coating with wide range of porosity (Figs. 6 vs. 8) and increased wickability of the coatings (Figs. 4c vs. 8d).

## Conclusions

A novel sintering technique with salt templating was developed in this work to achieve a large spectrum of pore diameters ranging from a few microns to a few hundred microns. The copper particle size used for sintering was optimized by comparing the performance with 1 µm, 20 µm, and 45 µm particles and the particles were draped with highly thermally conductive graphene nanoplatelets (GNP) using a ball milling technique. The combination of salt templating and GNP draping on optimized 20 µm copper particles was then implemented to develop the salt templated sintered surfaces. Amongst 2%, 3%, and 5 wt% GNP draping, the 3 wt% GNP draped surface yielded the highest CHF and HTC reported in the pool boiling studies involving graphene and porous coatings on a plain copper surface, with a CHF of 289 W/cm^2^ at a wall superheat of just 2.2 °C (HTC of 1,314 kW/m^2^ °C). This study showed that this extraordinary enhancement was achieved due to the combined effect of availability of wide ranged porous network, formation of interconnected pore pockets and tunnel structures, very high wickability owing to the GNP draping on copper particles. Therefore, designing the microscale surfaces with a large spectrum of pore diameters and draping of copper particles with highly thermally conductive GNP will be beneficial in further improving the performance.

## Methods

### Copper surfaces

The copper test surfaces similar to previous studies have been used in this work^26^. The copper test surfaces consist of a 17 mm × 17 mm area along with a central 10 mm × 10 mm boiling region. All the test surfaces are prepared using an oxygen free copper rod with a purity of 99.99%. The thermal conductivity of this section was 391 W/m K (± 9 W/m K). The copper rod was machined to achieve the desired dimensions using a computer based numerically controlled micro-milling process^19^. The surface roughness of the copper surfaces was then reduced to ~ 1.3 μm using a grinding operation. The boiling surfaces were cleaned with IPA and washed under the reflux of distilled water to eliminate any traces of the IPA. The bottom section of the test surface is comprised of a rectangular section which houses the three thermocouple holes to measure the surface temperature and the heat flux^30^.

### Ball milling

GNP and copper particles were dispersed in ethanol bath for 30 min to achieve a homogeneous mixture. The mixture was then ball milled at a rotation of 700 rpm for 1 h with ethanol acting as a process control agent. After every 15 min. of ball milling, ball mill machine was allowed to cool down for 1 h. Stainless steel balls were used and the ball-to-powder ratio of 40:1 was taken to ensure a formation of GNP reinforced copper powder. The homogeneous GNP-Cu powder was then coated on the copper heater surface using a sintering technique.

### Sintering technique

To generate the sintered surfaces, in this study, GNP draped copper powder was mixed with a sintering oil (commercially available screen-printing binder, Nazdar 9627) to create a paste. The binder-to-powder ratio of 1:2 was used in all the cases. To examine close control over the coating thickness, a single-pass screen printing technique was used. Initially, the sintering temperature was 450 °C for the duration of 30 min. to eliminate the binder from the coatings, and then was ramped up to 800 °C for a duration of 1 h in an inert helium atmosphere.

The mass ratio of ball milled GNP-Cu composite powder, sodium carbonate (particle size ~ 40–200 µm), and binder or sintering oil was 1:1/3:1/2 or 6:2:3. The complete mixture was then transferred to the plain copper surface using a screen-printing technique we developed in our previous work. These surfaces were then sintered at 800 °C for 1 h with an additional evaporation step of sintering oil at 450 °C for 30 min. Since the melting temperature of sodium carbonate is 851 °C and the maximum temperature during sintering was 800 °C, the particles were expected to remain in the coating. After sintering, the sintered surfaces were dipped in a distilled water bath for 2 h to dissolve sodium carbonate particles. Finally, the surfaces were washed under a reflux of distilled water to absolutely eliminate the traces of sodium carbonate. The thicknesses of sintered coatings were measured using Laser Confocal Microscope and average thickness of 65 ± 3 µm was obtained after sintering for all the coatings.

### Characterization

TESCAN Field Emission Mira III LMU (Czech Republic) and JSM-6400 V scanning electron microscope (SEM), JEOL, Ltd., Tokyo, Japan was used to observe the morphology and geometrical characteristics of pores of the sintered surfaces. The energy dispersive x-ray spectroscopy (EDS) analysis was performed on Bruker Quantax EDS with XFLASH 5010 detector attached to a field emission scanning electron microscope MIRA II LMH^30^. Transmission electron microscope JEOL 2010 in the range of 80 to 200 kV was used to observe the draping of GNP around copper particles. To confirm the successful deposition of GNP draped copper and to have a deeper understanding of the chemical composition on copper surface different characterization techniques were employed. A multi-wavelength Jobin Yvon Horriba LabRAM HR Raman Spectroscope using He–Ne Laser (λ = 632.8 nm) was considered to quantify the deposited GNP layers and the acquisition time of 10 s was used to capture the data. To produce repeatable and reliable data, a total of 10 scans were taken by observing the characteristic D and G peaks of graphene^30^. Additionally, static wettability and dynamic wicking of sintered surfaces were measured for the surfaces using a VCA Optima Goniometer contact angle measurement instrument. For dynamic wicking, a distilled water droplet of 2 µL was steadily brought into contact with the coated surfaces using a static sessile drop method and liquid propagation was recorded using a high-speed camera^28^.

### Sample preparation of GNP draped copper powder for TEM and SEM imaging

To prepare the samples for imaging, a small quantity of GNP draped copper powder was mixed in 20 mL water and then the solution was ultra-sonicated for 30 min. Eventually, after settling of heavy particles, a small quantity of non-agglomerated solution was withdrawn and transferred for imaging in transmission and scanning electron microscopes.

### Experimental setup

In this work, an experimental setup like Jaikumar and Kandlikar^21^ was used. The setup consisted a ceramic test chip holder, a glass water bath, and a heater block to supply the required heat to the test section. A detailed explanation of the test section and pool boiling test procedure is explained in the supporting information.

## Supplementary information

Supplementary Information 1.
