# Supplementary material for: Salt templated and graphene nanoplatelets draped copper (GNP-draped-Cu) composites for dramatic improvements in pool boiling heat transfer
Source: Sci Rep. 2020 Jul 20;10:11941. doi: 10.1038/s41598-020-68672-1 (PMC7371685; doi:10.1038/s41598-020-68672-1)
Supplement: Supplementary file 1 — Supplementary Information 1. [file 41598_2020_68672_MOESM1_ESM.docx]

**Supplementary Information**

**Salt Templated Graphene Nanoplatelets Draped Copper (GNP-draped-Cu) Composites for Dramatic Improvements in Pool Boiling Heat Transfer**

Aniket M. Rishi^1^, Satish G. Kandlikar^1, 2^ and Anju Gupta^3^ *

^1^Microsystems Engineering, Rochester Institute of Technology, 76 Lomb Memorial Drive, Rochester, NY 14623. E-mail: [amr6756@rit.edu](mailto:amr6756@rit.edu)

^2^Mechanical Engineering, Rochester Institute of Technology, 76 Lomb Memorial Drive, Rochester, NY 14623. E-mail: [sgkeme@rit.edu](mailto:sgkeme@rit.edu)

^3^Mechanical, Industrial and Manufacturing Engineering, University of Toledo, 2801 W. Bancroft St., Toledo, OH 43606. E-mail: [anju.gupta@utoledo.edu](mailto:Anju.gupta@utoledo.edu)

***Corresponding author:**

Anju Gupta

E-mail: [anju.gupta@utoledo.edu](mailto:anju.gupta@utoledo.edu)

Mechanical, Industrial and Manufacturing Engineering, University of Toledo, 2801 W. Bancroft St., Toledo, OH 43606.

Telephone: +1-(413)-530-8213

# Elemental analysis of GNP draped copper particles:

With increased wt. % of GNP, compared to 2% GNP, the increment in carbon concentration peak in the direction of red arrow was observed for 3% and 5% GNP draped particles (as observed from Fig. S1 a, b, and c). Additionally, we observed that the carbon peak intensity comparatively becomes stable, indicating that GNP are circumferentially deposited around the copper particles.


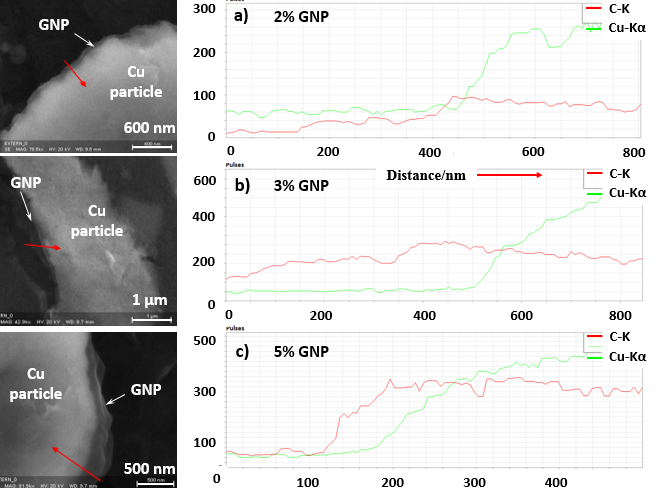


**Figure S1. Elemental analysis of GNP draped copper particles indicating the increment followed by saturation of carbon peaks in the direction of red arrow a) 2% GNP, b) 3% GNP, and c) 5% GNP**

# Characterization of coatings:

**Table 1. Test matrix for sintered and ball milled sintered composite coatings**

| **Coating technique** | **Test chip** | **Composition** |
| --- | --- | --- |
| Sintering | S1 | 1 µm Cu particles + 2 wt. % GNP |
|  | S2 | 20 µm Cu particles + 2 wt. % GNP |
| Draping via ball Milling followed by Sintering  (BM + sintered) | BM-S1 | 1 µm Cu particles + 2 wt. % GNP |
|  | BM-S2 | 20 µm Cu particles + 2 wt. % GNP |
|  | BM-S3 | 45 µm Cu particles + 2 wt. % GNP |

Figure S2 a) and S2 c) show the scanning electron microscope image with copper and carbon mapping after developing sintered coating of 1 µm Cu particles-2% GNP (S1) and 20 µm Cu particles-2% GNP (S2), respectively. Compared to 2% GNP-draped 1 µm Cu coating and 2% GNP-draped 20 µm Cu coating (Fig. S2 b) and S2 d)), carbon mapping on only sintered surfaces (Fig. S1 a) and S1 c)) indicate non-uniform GNP deposition on the heater surface. This confirms that homogeneous mixture is yielded after draping of GNP on Cu particles.


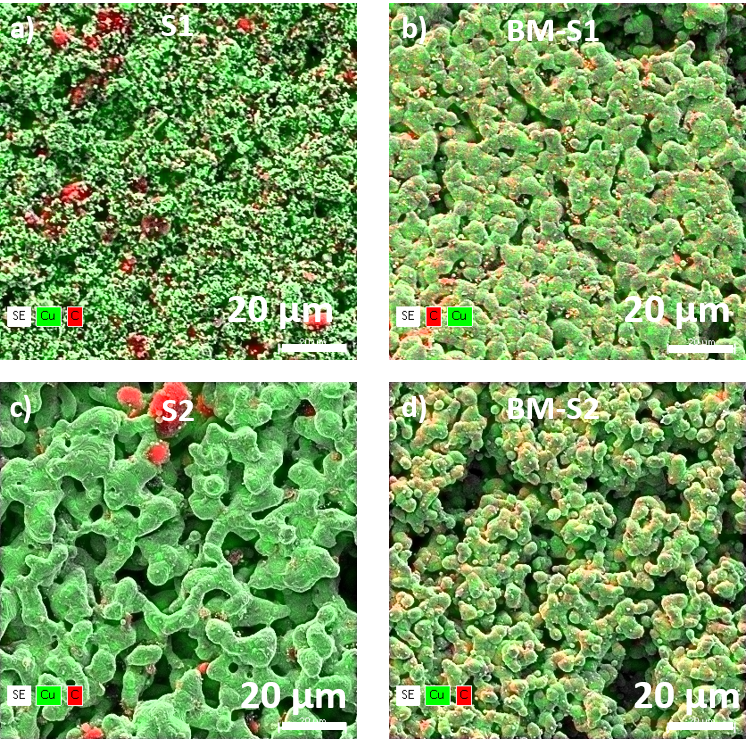


**Figure S2. Scanning electron microscope (SEM) images at 500X magnification of sintered surfaces showing carbon and copper mapping a) S1 – sintering of 1 µm Cu-2% GNP, b) BM-S1 – ball milling followed by sintering of 1 µm Cu-2% GNP, c) S2–sintering of 20 µm Cu-2% GNP, and d) BM-S2 – ball milling followed by sintering of 20 µm Cu-2% GNP**

Figure S3 compares the Figure S2 e) shows the Raman spectroscopy analysis of 2% GNP draped 1 µm, 20 µm, and 45 µm particle surfaces. As mentioned in the main manuscript, the ratios of intensities I_D_/I_G_ on Raman plot demonstrate the oxidation degree and defects and ratios I_G_/I_2D_ quantify the number of GNP layers in each sample. I_G_/I_2D_ ratio of around ~4 for all the GNP surfaces was observed indicating a multi-layer graphene deposition.


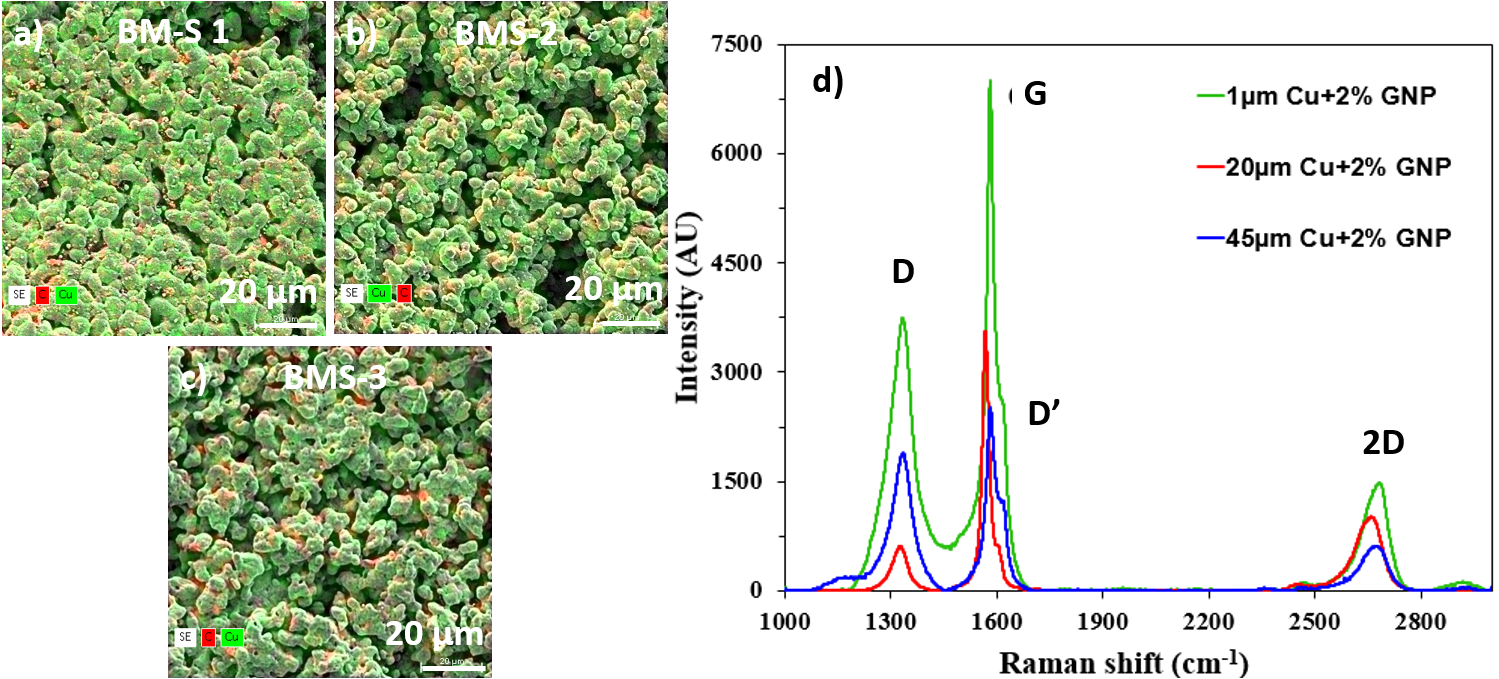


**Figure S3. Scanning electron microscope (SEM) images at 500X magnification of GNP draped copper particle surfaces showing carbon and copper mapping a) BM-S1 – 1 µm Cu-2% GNP, b) BM-S2 – 20 µm Cu-2% GNP, c) BM-S3 – 45 µm Cu-2% GNP, and d) comparison of Raman spectra of GNP draped copper particle surfaces**

Figure S4 shows the energy dispersive x-ray spectroscopy (EDS) plot indicating a peak intensity on y-axis and energy on x-axis. Figure S4 demonstrates a set of peaks only for carbon and copper based on their electromagnetic spectrum relevant to their unique atomic structures. This plot confirms that there is an absence of salt traces on the coating after washing under the reflux of distilled water. Also, with the corresponding increment in GNP wt. % from 2 to 5, increment in carbon peak intensity is observed.

Laser confocal microscope was used to generate a 3D micrograph for 20 µm Cu-3% GNP salt templated coating to demonstrate the formation of inhomogeneous coatings due to implementation of ball milled and salt templated sintering.


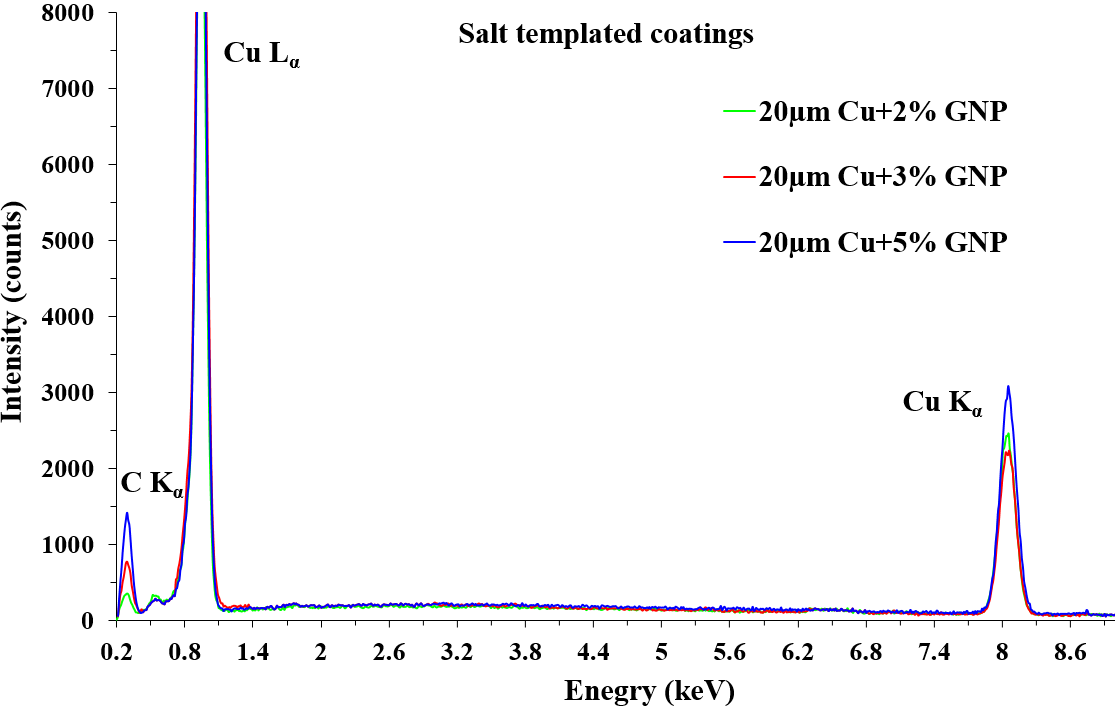


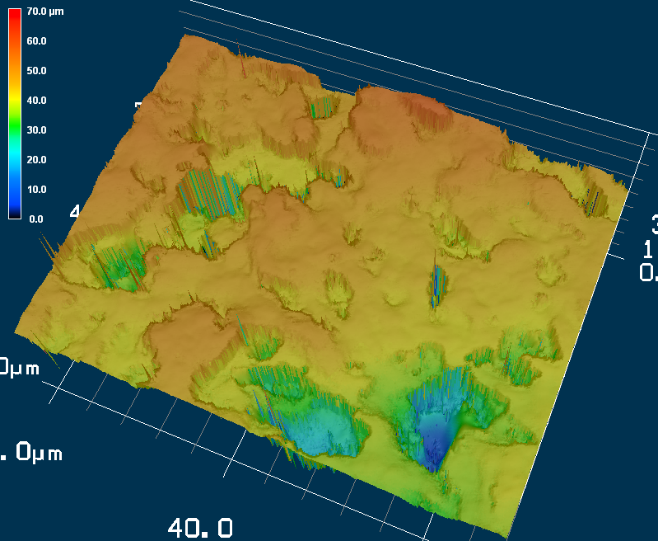


**Figure S4. a) Energy dispersive X-ray spectroscopy (EDS) analysis of the salt templated coatings, b) 3D micrograph of 20 µm Cu-3% GNP salt templated coating showing the inhomogeneous coating, captured using laser confocal microscope**

# Pool boiling test setup:

The pool boiling test setup used for all the experiments is shown in Figure S5. For heating the boiling surface and for the data acquisition, a plain copper test surface made from alloy 101 was manufactured using CNC machine that could measure the local temperatures and supply the heat, as shown in Figure S5. Four cartridge heaters (120 V-DC, 200 W capacity each) were inserted into a copper heater block and the 10 mm × 10 mm surface of the copper heater was kept in contact with the test surface, which also has a base section of 10 mm × 10 mm. This facilitated 1-D heat conduction from the heater to the test surface. Additionally, to minimize the heat losses, the copper heater block is housed on a ceramic sleeve. To monitor the temperatures, a National Instruments cDaq-9172 data acquisition system with an NI-9211 thermocouple input module was used. In addition, the graphical variation of temperature with respect to time for each K-type thermocouple was displayed through the LabVIEW interface. This was useful for determining the critical heat flux (CHF).


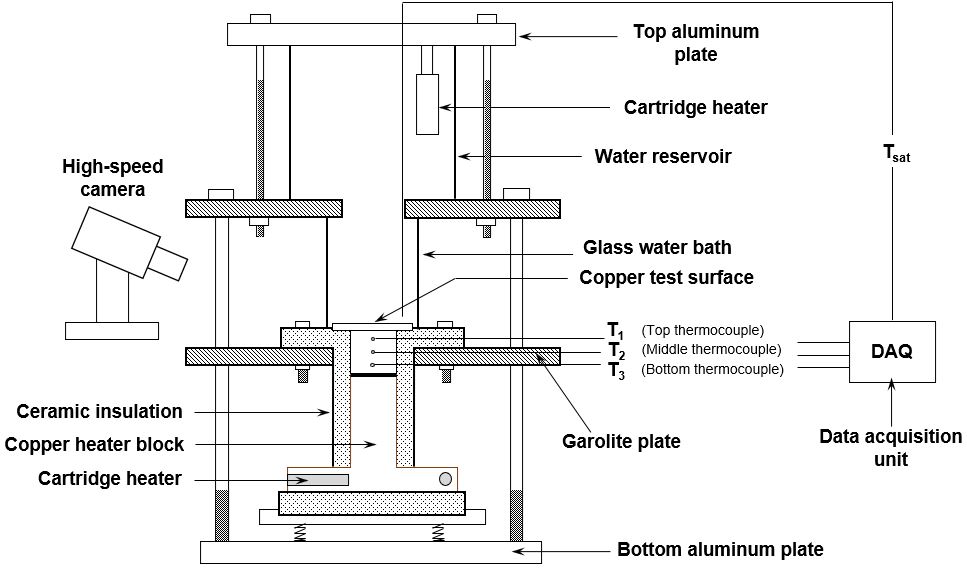


**Figure S5. Schematic of the pool boiling test system**

A bottom garolite plate with a ceramic chip holder was used to hold the test surface over which a quartz glass water bath was secured. A middle garolite plate holds the water bath on the upper side which is mounted between the middle garolite plate and a top aluminum plate. Rubber gaskets were used between the plates to make the setup leak proof. A top aluminum plate has two circular openings, one for inserting a thermocouple to measure the water temperature and the other for inserting a 60-VDC, 200 W auxiliary cartridge heater which maintains the water in the reservoir at saturation by boiling it continuously.

# Test Section and Data acquisition:

Plain test surfaces made of copper alloy 101 were used in this study. Test surfaces consisted of a 17 mm x 17 mm outer surface with an inner 10 mm square region providing the boiling surface. A 10-mm square stem protruded on the lower side as shown in Fig. S6 a). The lower section contacted the heater module. Three thermocouple holes were drilled to reach the center of the rectangular stem. The distances between the two successive holes in the rectangular stem is 3 mm (Δx) while the distance (x_1_) between the hole near the chip surface and the top of the chip is 1.5 mm. Figure S6 b) shows the boiling surface which was sintered for the pool boiling test.

To record the temperatures given by the thermocouples, a National Instruments cDaq-9172 data acquisition system with NI-9211 temperature module was used (as shown in Fig. S6-a). Overall four thermocouples were used, out of which three were inserted into the slots provided in the test section for heat flux measurement and surface temperature determination, while the fourth thermocouple was inserted from the top of aluminum block to measure the saturated temperature of the water. A LabVIEW software was used to show the measured temperatures from the thermocouples and it also showed the graphical variation of temperature with respect to time for each thermocouple which was then used to determine the critical heat flux spikes. A LabVIEW program was written which estimated the heat flux and the surface temperature values from the recorded temperature data.


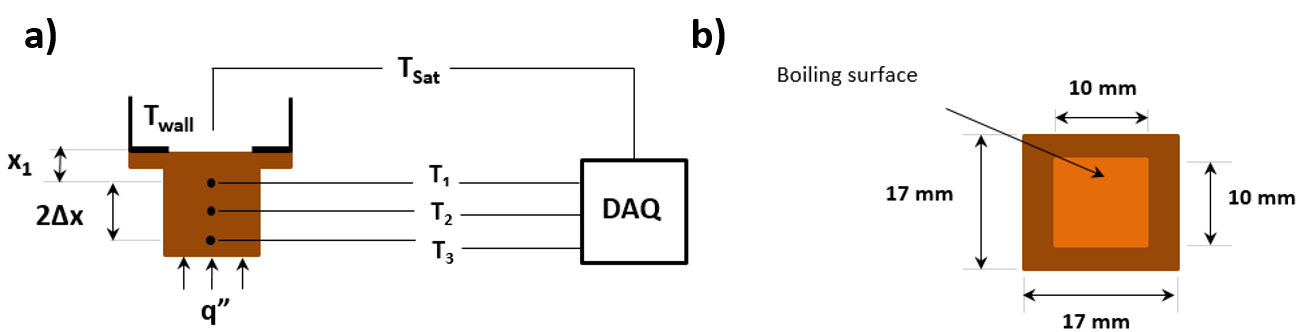


**Figure S6.** **Plain copper surface (a) data acquisition, (b) boiling surface**

Heat flux was calculated using steady state 1-D conduction equation:

$q^{''}= -k_{\mathrm{Cu}}\frac{\mathrm{dT}}{\mathrm{dx}}$ (1)

Where the temperature gradient $dT/dx$ was calculated using the three-point backward Taylor's series approximation

$\frac{\mathrm{dT}}{\mathrm{dx}}= \frac{3T_{1}-4T_{2}+T_{3}}{2\Delta x}$ (2)

The boiling surface temperature was obtained by using Eqs. 1 and 2, and is given by

$T_{\mathrm{wall}}= T_{1}- q^{''}\left( \frac{x_{1}}{k_{\mathrm{Cu}}} \right)$ (3)

The heat transfer coefficient for all the heat fluxes was calculated using Eq. 4.

$Heat transfer coefficient \left( HTC \right)= \frac{Heat flux (q")}{{Wall superheat temperature (\Delta T}_{sat})}$ (4)

# Uncertainty analysis:

The two main sources of errors in the experiments are precision errors and bias errors. Bias errors are the errors due to calibration while precision errors are due to the sensitivity of the testing instruments. Each thermocouple was calibrated over a temperature range higher than the testing temperature range. Based on the factors such as distance between thermocouples and the spacing between them, thermal conductivity of copper, and calibration data of thermocouples, the total uncertainty in heat flux and heat transfer coefficient were calculated.

The uncertainty in the heat flux and heat transfer coefficient can thus be expressed by the Eqs. 5 and 6 respectively.

$\frac{U_{q"}}{q"}= \sqrt{\left[ \left( \frac{U_{k}}{k} \right)^{2}+ \left( \frac{3U_{T_{1}}*k_{Cu}}{\Delta x*q"} \right)^{2}+ \left( \frac{4U_{T_{2}}* k_{Cu}}{\Delta x*q"} \right)^{2}+ \left( \frac{U_{T_{3}}* k_{Cu}}{\Delta x*q"} \right)^{2}+ \left( \frac{U_{\Delta x}}{\Delta x} \right)^{2} \right]}$(5)

$\frac{U_{h}}{h}= \sqrt{\frac{U_{q"}^{2}}{q^{"2}} +\frac{U_{T_{w}}^{2}}{{\Delta T}_{sat}^{2}}+ \frac{U_{T_{sat}}^{2}}{{\Delta T}_{sat}^{2}}}$ (6)

Where, $U_{q"}$ and $U_{h}$ are uncertainty in heat flux and heat transfer coefficient respectively. $U_{k}$ is the uncertainty in thermal conductivity, and $U_{T_{1}}$, $U_{T_{2}}$, $U_{T_{3}}$ are the uncertainties in temperature measurements in the thermocouples. The error analysis was obtained by the method of partial sums, which showed that the maximum uncertainty was from the thermocouple measurements. At higher heat fluxes, a maximum uncertainty of 4.6% in both CHF and HTC was obtained for the test surfaces.


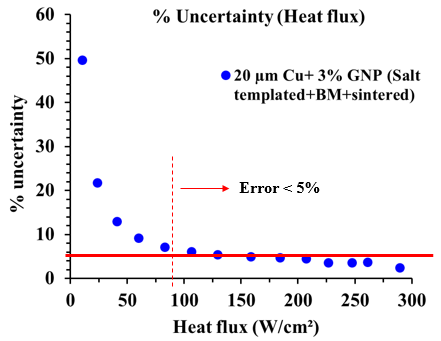

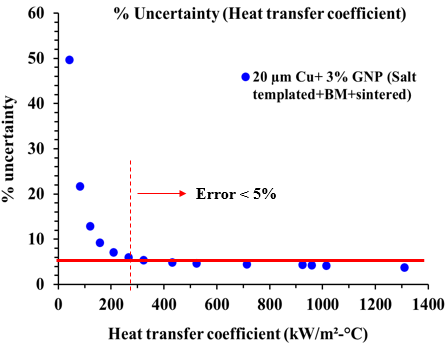


**Figure S7: % Uncertainty in heat flux and heat transfer coefficient for 20 um Cu+3% GNP salt templated and ball milled sintered coating**

**Effect of porous coatings on bubble dynamics:**


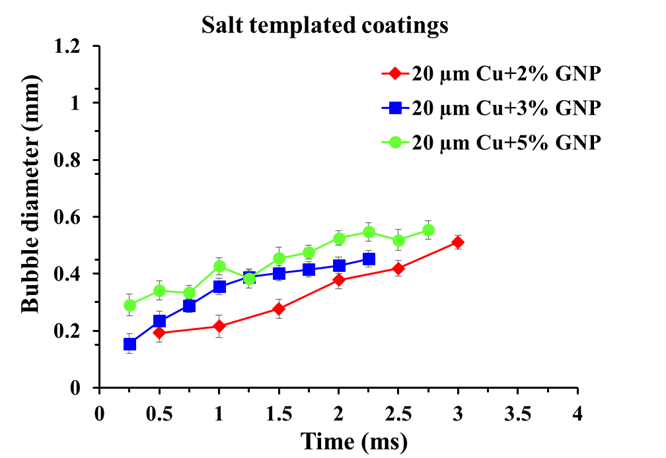


**Figure S8. Comparison of progression of bubble diameters w.r.t. time on different GNP wt. % salt templated coatings**

**Effect of repetitive pool boiling tests:**

Figure S9 below shows the pool boiling curve for the repetitive pool boiling tests where R1 represents repetitive pool boiling test 1 and so on. A significant deterioration of the pool boiling heat transfer performance was not observed indicating the strong adhesion between the coating and the substrate.

**
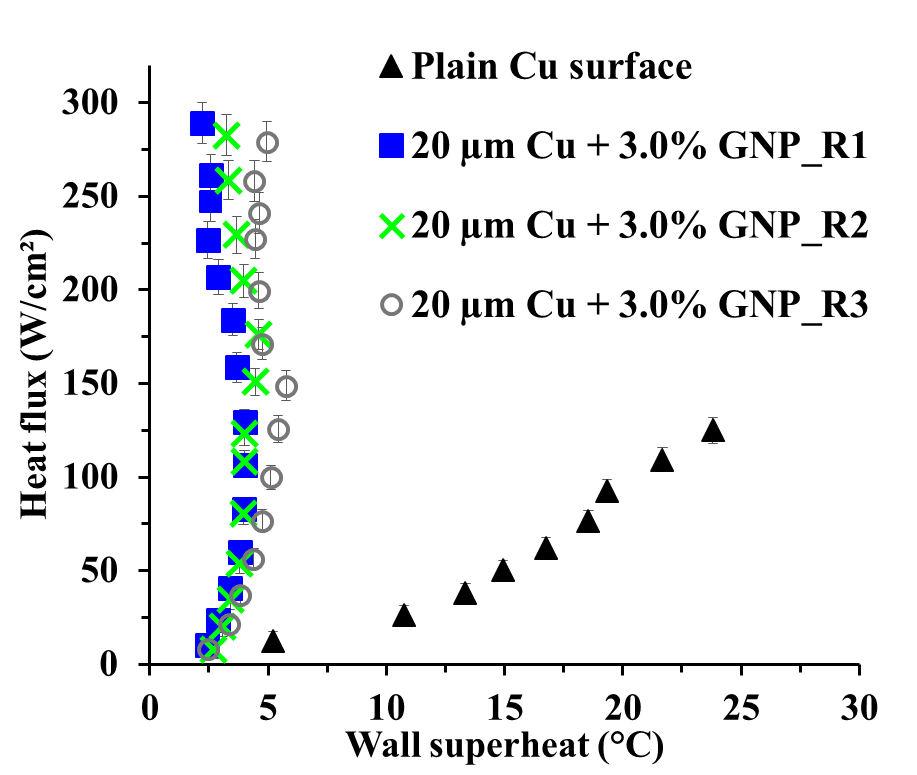
**

**Figure S9. Comparison of repetitive pool boiling performance on 20 µm Cu-3% GNP salt templated coating**
